# Comparison of GCaMP3 and GCaMP6f for studying astrocyte Ca^2+^ dynamics in the awake mouse brain

**DOI:** 10.1371/journal.pone.0181113

**Published:** 2017-07-24

**Authors:** Liang Ye, Mateen A. Haroon, Angelica Salinas, Martin Paukert

**Affiliations:** 1 Department of Cellular and Integrative Physiology, University of Texas Health San Antonio, San Antonio, TX, United States of America; 2 Xiangya School of Medicine, Central South University, Changsha, Hunan, China; 3 Center for Biomedical Neuroscience, University of Texas Health San Antonio, San Antonio, TX, United States of America; 4 Biomedical Engineering Joint Graduate Program, UTSA/UTHSA, San Antonio, TX, United States of America; Cinvestav-IPN, MEXICO

## Abstract

In recent years it has become increasingly clear that astrocytes play a much more active role in neural processes than the traditional view of them as supporting cells suggests. Although not electrically excitable, astrocytes exhibit diverse Ca^2+^ dynamics across spatial and temporal scales, more or less dependent on the animal's behavioral state. Ca^2+^ dynamics range from global elevations lasting multiple seconds encompassing the soma up to the finest processes, to short elevations restricted to so-called microdomains within fine processes. Investigations of astrocyte Ca^2+^ dynamics have particularly benefitted from the development of Genetically-Encoded Calcium Indicators (GECIs). GECI expression can be achieved non-invasively in a cell type-specific manner and it can be genetically targeted to subcellular domains. The GCaMP family, a group of GECIs derived from the green fluorescent protein, has experienced some of the fastest advancements during the past decade. As a consequence we are now facing the challenge of needing to compare published data obtained with different versions of GECIs. With the intention to provide some guidance, here we compared Ca^2+^ dynamics across scales in awake transgenic mice expressing either the well-established GCaMP3, or the increasingly popular GCaMP6f, specifically in astrocytes. We found that locomotion-induced global Ca^2+^ elevations in cortical astrocytes displayed only minor kinetic differences and their apparent dynamic ranges for Ca^2+^ sensing were not different. In contrast, Ca^2+^ waves in processes and microdomain Ca^2+^ transients were much more readily detectable with GCaMP6f. Our findings suggest that behavioral state-dependent global astrocyte Ca^2+^ responses can be studied with either GCaMP3 or GCaMP6f whereas the latter is more appropriate for studies of spatially restricted weak and fast Ca^2+^ dynamics.

## Introduction

Fluorescent Ca^2+^ indicators are an incredibly valuable tool for measuring neural activity. They enable localizing and monitoring activity levels in cell populations. Traditionally, chemical dyes have been employed. The first widely used dye, Fura-2, has been developed as a fast, ratiometric Ca^2+^ indicator that enables quantitative Ca^2+^ measurements [1]. Chemical Ca^2+^ indicators can be esterified with a lipophilic acetoxymethyl moiety that facilitates permeation of dyes through intact cell membranes and leads to accumulation of the dye following intracellular hydrolysis of the lipophilic moiety [2]. This approach has been extended to the bulk loading of hundreds of brain cells with chemical dyes in intact animals [3], and led to the first observations of Ca^2+^ dynamics in cortical astrocytes and cerebellar Bergmann glia in anesthetized and awake behaving mice [4,5]. However, bulk loading of chemical dyes has numerous drawbacks: it requires local injection of the loading buffer to the tissue under investigation and therefore is invasive and causes damage, loading is not cell type-specific, and sufficient dye concentration is maintained only for a couple of hours, precluding chronic experiments.

Genetically encoded Ca^2+^ indicators were sought as a method to overcome most of these drawbacks. Early approaches involved multiple-fluorophore sensors that rely on Förster resonance energy transfer (FRET). Calmodulin (CaM) is used as Ca^2+^ sensor that associates with the M13 domain from myosin light chain kinase when it has Ca^2+^ bound and induces a conformational rearrangement facilitating FRET between the fluorophores [6]. In 1999, Baird et al. developed circularly permutated (cp) fluorescent versions of green fluorescent protein (GFP) and the yellow and cyan versions YFP and CFP, in which the N- and C-terminal halves of the fluorophore were swapped and linked [7]. They were able to insert the protein calmodulin (CaM) into YFP with the result that Ca^2+^ binding increased the fluorescence intensity of YFP. The paper also suggested the same could be done for GFP. In 2001, Nakai and colleagues accomplished this task by combining cpGFP with the CaM/M13 strategy, developing the first version of GCaMP [8]. In this construct, Ca^2+^ induces conformational changes in CaM/M13, which prevents the solvent from reaching the chromophore, increasing fluorescence. The progress in GECI development made it possible to genetically target the molecule to specific tissues using viruses (lenti, adeno-associated) or transgenic mice [9]. The first generation GCaMP and other contemporary GECIs still had a number of drawbacks compared to synthetic Ca^2+^ dyes. The baseline fluorescence and sensitivity were much lower. The dynamic range (F_max_/F_min_) was narrow. The ratio of Ca^2+^ concentration to fluorescence was not linear. They were not reliably functional *in vivo* at physiological temperatures and pH. The kinetics were much slower, making it impossible to resolve individual action potentials. However, it had already been predicted that altering the amino acid sequence of GCaMP would change its properties significantly with the possibility of alleviating these problems. 2006 saw the creation of GCaMP2, a brighter and more stable version of GCaMP that made *in vivo* Ca^2+^ imaging of mouse cardiomyocytes possible [10]. Two years later the structure of GCaMP2 was resolved, which yielded insight into the mechanism of Ca^2+^ sensing and the associated change in fluorescence, and opened the gate towards rational design for further improvements [11,12]. A major advancement was realized with version GCaMP3, which had increased brightness, dynamic range, and affinity for Ca^2+^, making it possible to detect Ca^2+^ elevations caused by individual action potentials in vitro [13]. GCaMP3 was the first GECI version for which transgenic mice were generated that allowed non-invasive, global and chronic expression in a Cre recombinase-dependent manner [14,15]. The availability of these mice greatly propagated the utility of GCaMP3. By screening for beneficial mutations in the primary structure, a family of new versions of GCaMP were developed that provide even greater signal to noise ratios, giving more reliable and sensitive measures of neuronal activity [16]. They are called GCaMP 6s (slow), 6m (medium), and 6f (fast), based on the rise and decay kinetics. With improved kinetics, response amplitude and signal-to-noise ratio GCaMP6f has been the first GECI on par with Oregon Green BAPTA, one of the most popular chemical Ca^2+^ dyes. GCaMP 6s and 6m realized even larger improvements in response amplitude, at the cost of kinetics. Mice have also been generated for Cre recombinase-dependent expression of the GCaMP6 variants [17,18].

Astrocytes are not electrically excitable. As a consequence, with current technology, monitoring astrocyte Ca^2+^ dynamics represents the most accessible way of obtaining insight into their real-time functional state. Astrocytes undergo a wide range of Ca^2+^ dynamics regarding kinetics as well as spatial distribution within individual astrocytes as well as within the astrocyte population throughout the brain [19]. One extreme within this range of astrocyte Ca^2+^ dynamics is represented by global Ca^2+^ elevations that encompass the entire astrocyte, last longer than 5 s, and occur simultaneously in astrocytes in different regions of the brain [15]. This global astrocyte Ca^2+^ activation can be triggered by a mouse transitioning from a resting state to active locomotion [5,15] or by other forms of arousal, and depends on α_1_-adrenergic signaling [15,20]. Consistent with a role of noradrenergic signaling in arousal, attention and learning, noradrenergic signaling in hippocampal astrocytes has been reported to be involved in ATP release and subsequent weakening of glutamatergic synaptic transmission through a postsynaptic mechanism [21,22]. Similarly, it has been reported that noradrenergic signaling through cortical astrocytes leads to release of ATP and D-serine and is required for long term potentiation of neuronal responses [23]. Global Ca^2+^ activation of cortical astrocytes can also be induced with electrical stimulation of the nucleus basalis of Meynert, which leads to cortical acetylcholine release, and plays a role in cortical plasticity [24,25]. Cholinergic signaling through astrocytes in the hippocampus is involved in setting the inhibitory tone [26]. The other extreme within the range of astrocyte Ca^2+^ dynamics are spatially restricted (< 5 μm in diameter) and short (< 2 s) Ca^2+^ elevations within microdomains of the finest processes. These microdomain Ca^2+^ dynamics are only partially dependent on intracellular Ca^2+^ mobilization from the endoplasmic reticulum [27]. Ca^2+^ influx through the plasma membrane seems to play an important role for these faster and spatially restricted signals [28–31]. It has been proposed that astrocyte microdomain Ca^2+^ dynamics can cause vesicular release of glial signaling molecules, regulate plasma membrane neurotransmitter transport, and control the synthesis of membrane-permeable signaling molecules to influence neuronal excitability, synaptic strength and capillary blood flow [28,31–35]. Astrocyte Ca^2+^ elevations along individual processes may represent to varying degrees combinations of the mechanisms underlying global and microdomain Ca^2+^ dynamics.

The advantages of GCaMP3 for studying astrocyte Ca^2+^ dynamics together with the availability of transgenic mice to achieve non-invasive cell type-specific expression [14,15] has made this a very popular tool to study astrocytes [28,29,35–46]. With the advent of the more advanced sensor GCaMP6f, researchers are starting to migrate towards this new GECI [18,27,42]. As a consequence, we are now facing the challenge of needing to compare astrocyte data obtained with GCaMP6f to published GCaMP3 data acquired in different laboratories, using different equipment and protocols. To provide some aid to resolve this challenge, we sought to compare Ca^2+^ dynamics in cortical astrocytes of awake mice monitored with GCaMP3 or GCaMP6f using the same equipment and protocol. We studied global responses, microdomain Ca^2+^ dynamics, and Ca^2+^ waves in processes. We found that GCaMP3 and GCaMP6f were equivalent in many regards when measuring global responses, with GCaMP6f reporting the time course of Ca^2+^ transients more faithfully; however, GCaMP6f was considerably more sensitive in studies of microdomain Ca^2+^ dynamics and Ca^2+^ waves.

## Materials and methods

### Animals

Cre recombinase-conditional GCaMP3 [15], GCaMP6f [17] and tdTomato (Ai14) [47] mice were crossed to *GLAST-CreER* [15] mice to enable expression in astrocytes. GCaMP or tdTomato expression was induced by 3 intraperitoneal injections of 100 mg/kg body weight tamoxifen, dissolved in sunflower seed oil, within 5 days during the fourth postnatal week. For some experiments in S1 Fig we crossed GLAST-CreER(+/-);R26-lsl-GCaMP3(+/-) mice with IP3R2 knockout mice [48]. All experiments were conducted in accordance with National Institutes of Health guidelines and were approved by Institutional Animal Care and Use Committees at UT Health San Antonio.

### Chronic cranial window surgery

Craniotomies were performed in two steps. In the first surgery, a custom designed stainless steel plate was attached to the skull for immobilization of the head under the objective. In the second surgery, performed at least 3 days later, the bone was removed and replaced with a glass coverslip. Mice were anaesthetized by i.p. injection of ketamine (100 mg/kg) and xylazine (10 mg/kg). As soon as animals were unconscious, petroleum jelly was applied to the eyes. Following hair removal with Nair and skin disinfection with 70% ethanol, the scalp was incised and resected. The periosteum was then shaved off and approximately 3 mm of muscle surrounding the exposed skull was covered with a thin layer of cyanoacrylate cement. After drying, an 11 mm wide aluminum head plate with a 2 mm x 4 mm oval opening was centered above primary visual cortex V1 at lambda, 2.5 mm lateral from midline and attached to the skull using dental cement (C&B Metabond; Parkell Bio-Materials Div.). The second surgery was performed at least three days after mounting of the head plate under 1.5–2% vol./vol. isoflurane in O_2_. A 2.5 mm x 2.5 mm area of skull in the center of the opening was removed using a #12 scalpel blade. Three layers of No.1 cover glass, stacked with Norland Optical Adhesive #81 (Norland Products), replaced the skull and the edges were sealed with dental cement (Ortho-Jet Crystal, Lang Dental Manufacturing). Imaging was initiated at least two weeks after surgery. The time during recovery from the second surgery was used to habituate the mouse to the linear treadmill and the imaging environment.

### Two-photon microscopy

Fluorescence images were collected using a Movable Objective Microscope (MOM) (Sutter Instrument) equipped with a resonant scanner and a Nikon 16x, 0.8 NA objective. The microscope was controlled by a personal computer equipped with an Intel Core i7 CPU 4930 @ 3.4 GHz and 16 GB of RAM running ScanImage (v5.0) software (Pologruto et al., 2003). Acquired frames were 400 μm by 400 μm for locomotion-induced global Ca^2+^ dynamics and 50 μm by 50 μm for microdomain or process wave Ca^2+^ dynamics, both at 512 by 512 pixel resolution. Image acquisition rate was 30 frames/s. Two photon excitation was achieved using a Titanium:Sapphire laser (Chameleon Ultra II, Coherent) tuned to 920 nm and attenuated, so that an average power of 60 mW or less entered the brain. For signal detection with all experiments we employed H10770PA-40 GaAsP detectors (Hamamatsu) with the control voltage set to 530 mV, and DHPCA-100 high-speed current amplifiers (FEMTO) with the gain set to 10 kV/A. The head of the mouse was immobilized by attaching the head plate to a custom-machined stage mounted on the microscope table. Mice were kept on the stage for a maximum of two hours.

### Locomotion paradigm

Head immobilized mice were placed on a custom designed linear treadmill. The treadmill was either freely movable so that animals could move at will, or under motor control. The motion of the belt of the treadmill was monitored with a mechanically coupled optical encoder. The signal of the optical encoder was digitized at 20 kHz simultaneously with the position signal of the slow scan mirror (custom-written LabVIEW routines controlling PXIe-6363, National Instruments) for post hoc determination of movement velocity during corresponding images.

### Electromyography

Body surface potential differences were recorded as the voltage between two silver wires placed subcutaneously at the right shoulder and left hip using an EXT-02 B amplifier (npi electronic). Data were digitized at 20 kHz (custom-written LabVIEW routines controlling PXIe-6363, National Instruments) for post hoc analysis. Muscle activity was extracted by applying a fast Fourier transform to the data and determining the power in the range 200 Hz—1 kHz. Fold increase was determined as power / power_Baseline_.

### Visual stimulation

A UV-LED (UVTOP-355-TO39-FW, Sensor Electronic Technology Inc.) with a Lambertian emission profile was used as a light source at a distance of 40 mm centered between the eyes to achieve uniform light exposure. The light power entering each eye with a pupil diameter of 2 mm was 7 nW. To eliminate optical cross talk between visual stimulation and two-photon fluorescence detection, the objective was shielded from the light source.

### Data analysis—Locomotion-induced global Ca^2+^ elevations

Data were processed and analyzed in MATLAB using built-in functions integrated into custom routines. Images were first processed with a spatial Gaussian filter (1.52 SD per pixel distance) to reduce stochastic noise of the detector. To compensate for motion artifacts during image series, we acquired weak autofluorescence signals through the red detection path as reference signal to the simultaneously acquired GCaMP signal through the green detection path. Whole-frame normalized 2D cross-correlation (built-in function “normxcorr2”) was determined for reference images, and individual frames were registered to maximize correlation. The same registration parameters were then applied to images of GCaMP fluorescence. Data were temporally averaged by a factor of 6, preserving a frame rate of 5 Hz. ΔF/F fluorescence intensity ("Ca^2+^ change") traces represent (F—F_median_) / F_median_ with F representing mean fluorescence value of all pixels within a region of interest (ROI) of one image frame and F_median_ representing median F of all image frames before the locomotion event. ROIs were the thresholded area of an image frame distinguishing GCaMP expressing astrocytes from background. Quantification of individual Ca^2+^ responses represents mean ΔF/F within 2–12 s following the onset of locomotion. The peak amplitude of calcium response was the point of maximal Ca^2+^ ΔF/F during a trial. Quantification of the time course of Ca^2+^ responses was done by calculating the rate of change in fluorescence intensity 10 seconds before and after the peak following locomotion. Area under the curve was determined by integrating the normalized ΔF/F 10 seconds before and after the peak. Trials in which Ca^2+^ changes resulting from voluntary locomotion or startle responses before or after enforced locomotion exceeded 9% ΔF/F were excluded to avoid distortion of the time course of enforced locomotion-induced fluorescence changes.

### Data analysis—Spontaneous microdomain Ca^2+^ dynamics

Two-photon image series covering 50 μm by 50 μm and lasting 100 s were processed as described for global Ca^2+^ data up to the temporal averaging step. We then eliminated all episodes of image frames which contained a global astrocyte Ca^2+^ elevation. Spatial binning of the original 512 by 512 pixel image frames was applied to achieve a final pixel size of 1.56 μm. For each pixel we applied an averaging filter using the mean of that pixel value in four consecutive image frames. We then calculated the differential signal (difference of any pixel value between two consecutive image frames) and filtered that differential signal as described above. A search for the threshold which identified the largest number of solitary or pairs of supra-threshold pixels followed, since microdomain Ca^2+^ dynamics are spatially restricted to less than 3 μm. Despite our finding of significantly more frequent microdomain Ca^2+^ events with GCaMP6f than with GCaMP3 (see Results), the thresholds that yielded the highest number of microdomain locations was not different (GCaMP3: 12.6 ± 1.1% ΔF/F; 9 mice—61 astrocytes; GCaMP6f: 10.1 ± 1.7% ΔF/F; 11 mice—82 astrocytes; p = 0.253). Once we had identified the locations (pixels or pixel pairs) of microdomain Ca^2+^ events, we counted all events that occurred during the observation period and calculated the frequency.

### Data analysis—Spontaneous Ca^2+^ waves along processes

For analysis of Ca^2+^ waves along processes (non-global Ca^2+^ dynamics that sequentially encompass more than 3 μm), we used the image series described for microdomain Ca^2+^ dynamics following the spatial binning. Using MATLAB, we calculated the mean of the Pearson's linear correlation coefficients between all pairs of image frames of an astrocyte. The rationale for this analysis was that the microdomain Ca^2+^ events, which are short and encompass only 1–2 of the up to 1024 pixels of an image frame in this analysis, will have a minor influence on image variability. On the other hand, following image registration and removal of global Ca^2+^ elevations, Ca^2+^ waves along astrocyte processes will dominate variability among fluorescence images. For imaging tdTomato fluorescence in V1 astrocytes, we applied 50–70 mW of 800 nm at the window surface and employed all other imaging and analysis procedures as described above for GCaMP experiments.

### Statistical analysis

For all locomotion-induced Ca^2+^ data sets, measurements from individual astrocytes within one 400 μm by 400 μm field of view per mouse were averaged. For all microdomain Ca^2+^ dynamics or Ca^2+^ waves, the analysis results from 3–10 astrocytes per mouse were averaged. In all cases, the statistical power was based on the number of mice. We first conducted a Lilliefors test to determine if statistical tests for normally or non-normally distributed populations were applicable. We used the unpaired Student's t-test for normally distributed data sets, and we used the Kruskal-Wallis test for non-normally distributed data sets, as indicated in the figure captions. For S1 Fig we used one-way ANOVA followed by Bonferroni correction for multiple comparisons. For all tests, p values smaller than 0.05 were considered to indicate significance. All data values have been summarized in S1 File with explanations of the data organization in S2 File.

## Results

**Fig 1:**
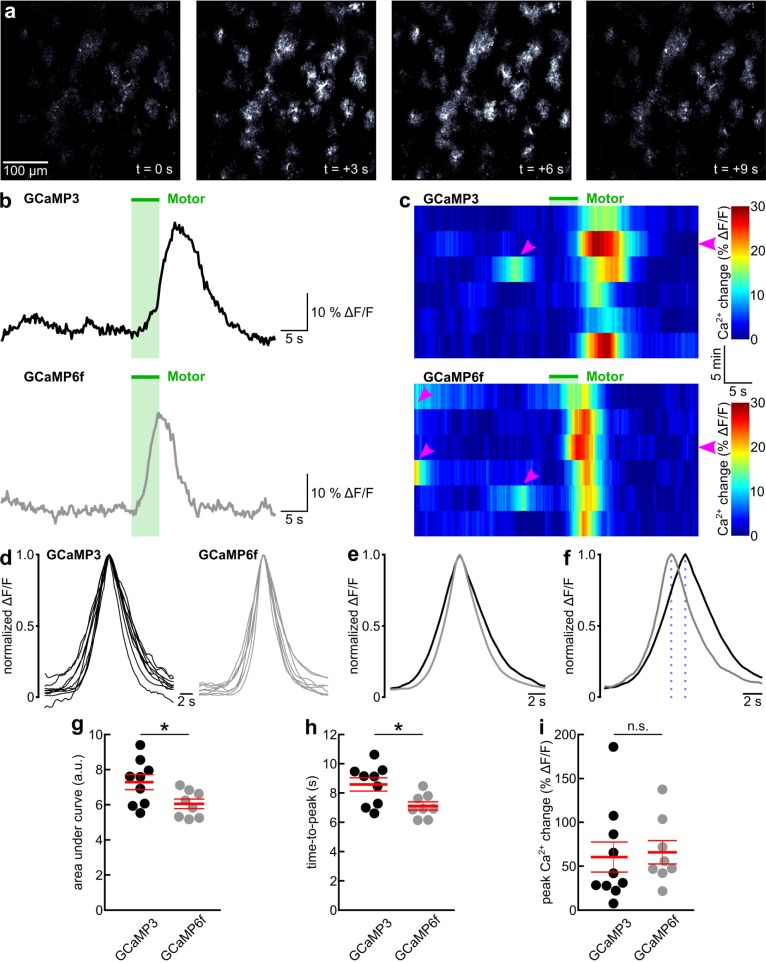
GCaMP6f indicates faster kinetics of astrocyte Ca^2+^ dynamics than GCaMP3. (**a**) Series of representative fluorescence images of astrocytes in layer 1 of primary visual cortex of an awake GLAST-CreER(+/-);R26-lsl-GCaMP6f(+/-) mouse. Image series covers one 5 s enforced locomotion event. (**b**) Black and gray traces represent time course of mean GCaMP3 or GCaMP6f fluorescence, respectively, from population of astrocytes as shown in a, in response to enforced locomotion (green bar). (**c**) Pseudocolored time course of GCaMP3 or GCaMP6f fluorescence in astrocytes in response to 6 consecutive trials of enforced locomotion (green bar). Flat magenta arrowheads indicate trials presented in b. Angled magenta arrowheads highlight prominent fluorescence transients in response to voluntary locomotion events. (**d**) Overlay of peak-aligned and normalized representative GCaMP3 (9 mice) or GCaMP6f (8 mice) fluorescence traces in response to enforced locomotion. (**e**) Overlay of peak-aligned and normalized fluorescence traces in response to enforced locomotion from astrocytes expressing GCaMP3 (black trace, 9 mice) or GCaMP6f (gray trace, 8 mice). (**f**) Same traces as in e, time-shifted to represent mean time from onset of enforced locomotion to peak (highlighted by dotted lines) of GCaMP3 (black trace) or GCaMP6f (gray trace) fluorescence. (**g**) Population data representing area under enforced locomotion-induced GCaMP fluorescence transient shown in e. a.u. represents seconds multiplied by normalized ΔF/F. Asterisk indicates p = 0.032 (unpaired t-test). Red lines represent mean ± SEM. (**h**) Population data representing time from onset of enforced locomotion to peak of GCaMP fluorescence shown in f. Asterisk indicates p = 0.017 (unpaired t-test). Red lines represent mean ± SEM. (i) Population data representing peak amplitudes of enforced locomotion-induced astrocyte GCaMP fluorescence transients. GCaMP3, 10 mice; GCaMP6f, 8 mice; n.s. indicates p = 0.813 (unpaired t-test). Red lines represent mean ± SEM.

For studying astrocyte-specific Ca^2+^ dynamics we used adult (2–6 months old) transgenic mice heterozygous for GLAST-CreERT and R26-lsl-GCaMP3 [15] or R26-lsl-GCaMP6f [17]. Tamoxifen treatment induced astrocyte-specific expression of GCaMP3 (not shown; [15]) or GCaMP6f (Fig 1A) in primary visual cortex.

### Locomotion-induced global astrocyte Ca^2+^ elevations

**Fig 2:**
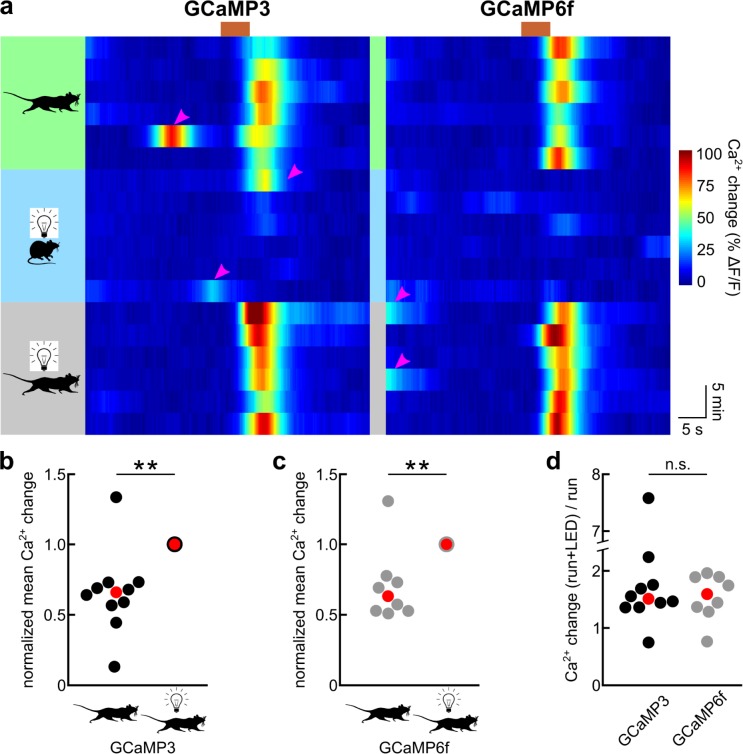
GCaMP3 and GCaMP6f operate with a similar dynamic range in astrocytes. (**a**) Pseudocolored time course of primary visual cortex astrocyte GCaMP fluorescence in 6 consecutive trials of enforced locomotion (green field), visual stimulation (blue field) or enforced locomotion combined with visual stimulation (gray field) during the time window indicated by the brown bar. Magenta arrowheads highlight prominent fluorescence transients in response to voluntary locomotion events. (**b**) Population data representing mean GCaMP3 fluorescence increases in response to enforced locomotion normalized to responses to enforced locomotion with simultaneous visual stimulation in the same experimental session (10 mice; asterisks indicate p = 0.001 (Kruskal-Wallis test)). Red symbol indicates median. (**c**) Population data representing mean GCaMP6f fluorescence increases in response to enforced locomotion normalized to responses to enforced locomotion with simultaneous visual stimulation in the same experimental session (8 mice; asterisks indicate p = 0.007 (Kruskal-Wallis test)). Red symbol indicates median. (**d**) Population data representing fold increase of locomotion-induced mean GCaMP fluorescence increases by simultaneous visual stimulation (n.s. indicates p = 0.859 (Kruskal-Wallis test)). Red symbol indicates median.

For a quantitative comparison of GCaMP3 and GCaMP6f fluorescence signals in response to astrocyte Ca^2+^ dynamics in awake behaving mice, we employed the enforced locomotion of head-fixed mice on a linear treadmill, a paradigm that we previously developed [15]. For behavioral state-dependent global Ca^2+^ dynamics, we acquired image series of 3–29 primary visual cortex astrocytes in one 400 μm by 400 μm field of view per mouse at a rate of 5 images per second (after posthoc averaging). Both GCaMP3 as well as GCaMP6f underwent robust, slowly rising fluorescence increases in astrocyte somata and processes lasting longer than 5 s in response to a 5 s enforced locomotion event (Fig 1A–1C). Facilitated by the well-controlled enforced locomotion stimulus, we could quantify three functional parameters of GCaMP3 and GCaMP6f responses to locomotion-induced global Ca^2+^ elevation: kinetics, amplitude and dynamic range. For the comparison of GCaMP response kinetics, we normalized locomotion-induced fluorescence increases and temporally aligned them to the response peak (Fig 1D). The area under the normalized fluorescence transient, as a compound parameter controlled by rise and decay, was smaller with GCaMP6f (6.05 ± 0.27 s * normalized ΔF/F; 8 mice) than with GCaMP3 (7.28 ± 0.43 s * normalized ΔF/F; 9 mice, p = 0.032) (Fig 1E and 1G). This finding suggests faster kinetics of GCaMP6f responses to global astrocyte Ca^2+^ elevations. Indeed, GCaMP6f fluorescent transients had a larger maximum rate of rise (35.2 ± 2.0% of peak / s; 8 mice) than GCaMP3 fluorescent transients (27.9 ± 1.8% of peak / s; 9 mice, p = 0.014), and there was a trend towards a faster maximum rate of fluorescence decay of GCaMP6f signals (-26.2 ± 2.1% of peak / s) compared with GCaMP3 signals (-21.6 ± 1.3% of peak / s; p = 0.080). Consistent with these kinetic parameters, GCaMP6f signals reached the peak response sooner following onset of locomotion (7.11 ± 0.29 s; 8 mice) than GCaMP3 signals (8.58 ± 0.45 s; 9 mice, p = 0.017) (Fig 1F and 1H). In contrast, the peak amplitude of fluorescence change over baseline reached following enforced locomotion was not different (GCaMP3: 60.33 ± 16.19% ΔF/F, 10 mice; GCaMP6f: 65.75 ± 13.29% ΔF/F, 8 mice; p = 0.813) (Fig 1I). Changes in sensor kinetics are often linked to changes in affinity for the ligand, with faster decay kinetics usually being indicative of a lower affinity [49]. Therefore, we sought to compare the dynamic ranges for Ca^2+^ sensing of GCaMP3 and GCaMP6f expressed in astrocytes. Ideally, we would like to determine the Ca^2+^ concentration—fluorescence relationship for each of the two GCaMPs. However, given the constraints of awake mouse experiments, we sought to compare the respective ratio of fluorescence responses to two different Ca^2+^ concentrations within the dynamic range of the sensor. We previously found that locomotion-induced Ca^2+^ elevations in primary visual cortex astrocytes can be potentiated by simultaneous visual stimulation, and that visual stimulation alone is not sufficient to cause a global astrocyte Ca^2+^ response [15]. This observation indicates that locomotion-induced astrocyte Ca^2+^ elevations in primary visual cortex do not saturate GCaMP3. We confirmed this observation here, as locomotion-induced GCaMP3 fluorescence was approximately two thirds the size reached with combined locomotion and visual stimulation (GCaMP3 response_locomotion_ normalized to GCaMP3 response_locomotion+LED_: 0.66 ± 0.09, 10 mice; p = 0.001) (Fig 2A and 2B). Like GCaMP3 (Fig 2A) [15], GCaMP6f did not indicate global primary visual cortex astrocyte Ca^2+^ elevations in response to visual stimulation when mice were at rest (Fig 2A). Similarly, GCaMP6f-mediated fluorescence increases to locomotion were smaller than responses to combined locomotion and visual stimulation (GCaMP6f response_locomotion_ normalized to GCaMP6f response_locomotion+LED_: 0.71 ± 0.09, 8 mice; p = 0.007) (Fig 2A). If GCaMP6f had a similar apparent Ca^2+^ affinity in astrocytes as GCaMP3, we would predict that they would have a similar ratio of responses to simultaneous locomotion and visual stimulation over responses to locomotion alone. Indeed, simultaneous visual stimulation potentiated locomotion-induced GCaMP6f responses similarly strongly as with GCaMP3 (GCaMP3: median fold potentiation: 1.51x (range: 0.75x - 7.58x), 10 mice; GCaMP6f: median fold potentiation: 1.59x (range: 0.76x - 1.96x), 8 mice; p = 0.859) (Fig 2D). Together, these data indicate that GCaMP6f detects astrocyte Ca^2+^ dynamics with moderately faster kinetics while preserving the useful dynamic range of GCaMP3 for sensing behavioral state-dependent global astrocyte Ca^2+^ elevations. For these slow global Ca^2+^ transients the kinetic improvements with GCaMP6f did not translate into increased signal amplitudes.

### Spontaneous astrocyte microdomain Ca^2+^ dynamics and Ca^2+^ waves

**Fig 3:**
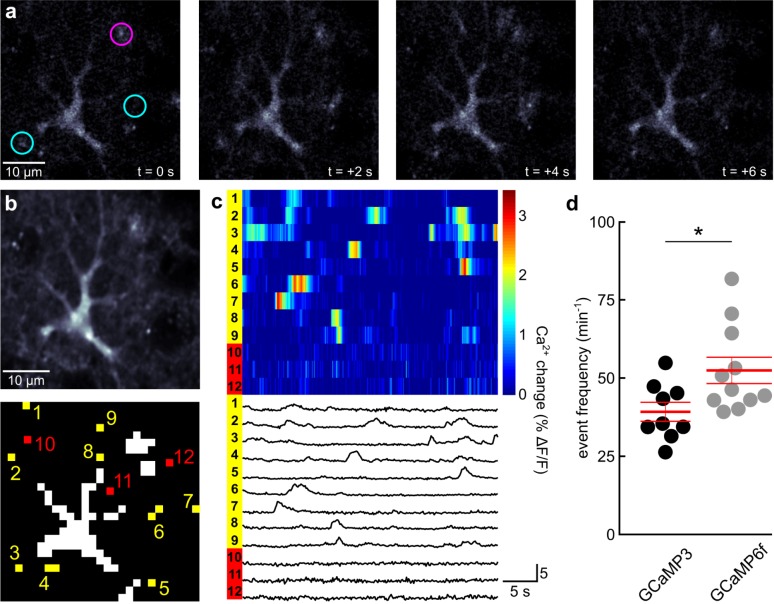
Astrocyte microdomain Ca^2+^ fluctuations can be detected more readily with GCaMP6f than with GCaMP3. (**a**) Series of representative fluorescence images of an astrocyte in layer 1 of primary visual cortex of an awake GLAST-CreER(+/-);R26-lsl-GCaMP6f(+/-) mouse. Magenta circle highlights area of persistently enhanced fluorescence. In contrast, cyan circles highlight areas of transient, spatially restricted fluorescence increases, typical for microdomain Ca^2+^ fluctuations. (**b**) upper, Average of 250 consecutive astrocyte images, as shown in a. lower, Clusters with not more than two suprathreshold pixels during a 100 s time window are highlighted in yellow (1–9) and are considered locations of microdomain Ca^2+^ fluctuations. Example pixels representing areas of the astrocyte process tree without suprathreshold events during the observation time window are highlighted in red (10–12). (**c**) **upper**, Pseudocolored time course of GCaMP6f fluorescence at 12 labeled locations highlighted in b. **lower**, corresponding fluorescence traces. (**d**) Population data of frequency of detected microdomain Ca^2+^ fluctuations in mice expressing GCaMP3 (9 mice—61 astrocytes) or GCaMP6f (11 mice—82 astrocytes). Asterisk indicates p = 0.025 (unpaired t-test). Red lines represent mean ± SEM.

**Fig 4:**
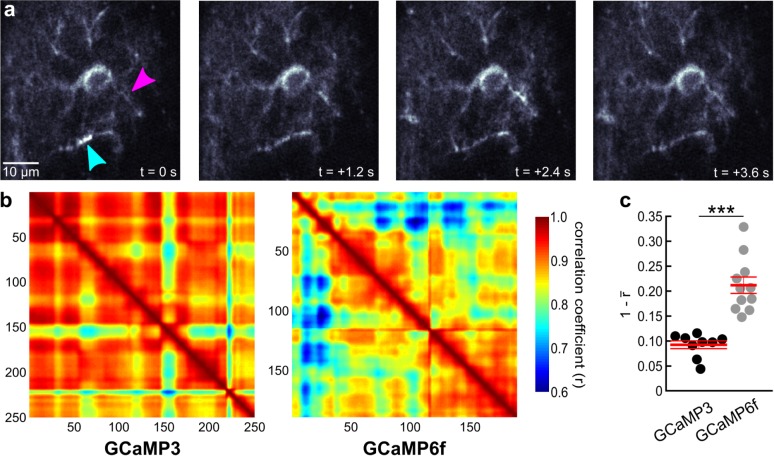
Calcium waves along astrocyte processes can be detected more readily with GCaMP6f than with GCaMP3. (**a**) Series of representative fluorescence images of an astrocyte in layer 1 of primary visual cortex of an awake GLAST-CreER(+/-);R26-lsl-GCaMP6f(+/-) mouse. Magenta arrowhead highlights region where a full wave cycle was captured, cyan arrowhead highlights a region where the decaying phase of a wave was covered. (**b**) Representative pairwise Pearson correlation coefficient plots of series of indicated number of fluorescence images. Cooler colors indicate lower correlation and more dynamic GCaMP indication of Ca^2+^ fluctuations. (**c**) Population data of 1—mean correlation coefficient (r). GCaMP3, n = 9 mice (61 cells) and GCaMP6f, n = 11 mice (82 cells); asterisks indicate p < 0.001 (unpaired t-test). Red lines represent mean ± SEM.

The faster kinetics of GCaMP6f signaling suggested benefits for the detection of shorter Ca^2+^ transients, such as in spatially restricted microdomains of fine astrocyte processes (Fig 3A). We acquired image series at 5 image frames per second (after posthoc averaging) for 100 s per astrocyte. Following removal of images with a global astrocyte Ca^2+^ elevation, we applied a fully automatic analysis algorithm for unbiased quantification of microdomain Ca^2+^ dynamics. The analysis algorithm (see Materials and Methods for details) searched for the threshold that yielded the largest number of individual size-restricted supra-threshold domains and determined the number of fluorescence transients that occurred at the detected domains during the time interval of observance (Fig 3B). As expected for microdomain Ca^2+^ dynamics, fluorescence transients were short (less than 5 s) and occurred in an asynchronous, uncoordinated manner (Fig 3C). We found that GCaMP6f made it possible to detect microdomain Ca^2+^ events in individual astrocytes of resting, awake mice more frequently (52.43 ± 4.20 events / s; 11 mice—82 astrocytes) than when imaged using GCaMP3 (39.21 ± 3.02 events / s; 9 mice—61 astrocytes, p = 0.025). Astrocytes also exhibit intermediate, wave-like Ca^2+^ dynamics along individual processes. Since Ca^2+^ waves are constituted by a spatial and temporal sequence of short Ca^2+^ transients at any location along a process (Fig 4A), we hypothesized that GCaMP6f might also prove to be more sensitive for the detection of astrocyte subcellular Ca^2+^ waves. For quantifying astrocyte Ca^2+^ waves in a completely unbiased way, we reasoned that the highly variable pattern of Ca^2+^ waves that can encompass considerable fractions of the astrocyte processes would reduce the average cross-correlation among frames of an image series (Fig 4B). We first tested whether this analysis preferentially reflects Ca^2+^ wave activity in contrast to microdomain Ca^2+^ events. Ca^2+^ waves, like global astrocyte Ca^2+^ elevations, are strongly reduced in inositol trisphosphate receptor 2 (IP3R2) knockout mice, whereas microdomain Ca^2+^ events are smaller but not less frequent [27]. Consistent with the notion that image cross-correlation analysis is not sensitive to microdomain Ca^2+^ dynamics, the distance to a perfect correlation of 1 was not significantly different when a Ca^2+^ unresponsive tdTomato signal was analyzed (0.04 ± 0.01; 6 mice) compared to the GCaMP3 signal in IP3R2 (-/-) mice (0.05 ± 0.01; 8 mice; S1 Fig). In contrast, image series acquired with GCaMP3 in wildtype mice revealed a considerably larger distance to a perfect correlation of 1 (0.09 ± 0.01; 9 mice; Fig 4C and S1 Fig) compared to tdTomato mice (p < 0.05—ANOVA). Importantly, image series acquired in wildtype mice using GCaMP6f revealed a much larger distance to a perfect correlation of 1 (0.21 ± 0.02; 11 mice, p < 0.001—unpaired t-test) than with GCaMP3 (Fig 4C and S1 Fig) Together, these data indicate that, compared with GCaMP3, GCaMP6f is more sensitive to the detection of fast astrocyte Ca^2+^ dynamics at small process terminals, or of Ca^2+^ waves along processes.

## Discussion

Our investigations revealed that GCaMP6f was more sensitive than GCaMP3 in the detection of small and fast microdomain Ca^2+^ dynamics in cortical astrocytes of awake mice, as well as in the detection of Ca^2+^ waves along astrocyte processes. In contrast, there was no difference in the amplitude of GCaMP6f or GCaMP3 fluorescence changes in response to locomotion-induced slow, global Ca^2+^ elevations. While these findings appear contradictory at first glance, they are more conclusive when we consider the properties of these GECIs in more detail. GCaMP6f has been developed from GCaMP5G, which in turn was derived from GCaMP3 [16,37]. GCaMP5G carries three point mutations within the GCaMP3 substrate. Two amino acid substitutions (T302L, R303P) in the linker 2 region, which connects cpEGFP with CaM, lead to an increased dynamic range of Ca^2+^-induced fluorescence increase at the cost of Ca^2+^ affinity. A third mutation (D380Y) within the interlobe linker of CaM restitutes Ca^2+^ affinity and favors low fluorescence signals of GCaMP5G in the absence of Ca^2+^ at physiological pH. The latter property is critical for the extended dynamic range of fluorescence change of this indicator during *in vivo* experiments [37]. The GCaMP6 family of GECIs carries a common set of three additional single amino acid substitutions. Two (T381R, S383T) are located in the interlobe linker of CaM and may facilitate anchoring the Ca^2+^ bound CaM-M13 complex to the cpEGFP, thereby controlling solvent access to the chromophore and controlling its protonation state, accounting for the increased Ca^2+^-dependent fluorescence increase [50]. The third mutation (R392G) was adapted from the K version of GCaMP5; it is located close to one of the CaM Ca^2+^ binding sites [50] and makes GCaMP5K the GCaMP5 with the highest Ca^2+^ affinity [51]. GCaMP6f is the fastest GCaMP due to an additional mutation (A317E) in a region of CaM that is involved in the interaction with the M13 peptide. The substitution of the short hydrophobic alanine with the long, charged glutamate is thought to destabilize the Ca^2+^-induced CaM-M13 complex formation [50]. Remarkably, the considerably enhanced Ca^2+^ sensitivity of GCaMP6f in cultured hippocampal neurons compared to GCaMP3 is predominantly noticeable with small and short Ca^2+^ elevations. For example, GCaMP6f fluorescence increases in response to 1–2 action potentials are up to 10 times larger than when imaged with GCaMP3 [16]. However, with increasing stimulus strength this benefit fades away, and with a burst of 100 action potentials GCaMP6f fluorescence increases are some twofold as large as GCaMP3 responses. Locomotion and startle-induced astrocyte Ca^2+^ elevations depend on α_1_-adrenergic signaling [15,20], Ca^2+^ transients encompass the entire cell and display a time course (>10 s) consistent with G_q_-coupled receptor mediated intracellular Ca^2+^ release. Therefore, our finding that GCaMP6f and GCaMP3 indicate locomotion-induced Ca^2+^ elevations equally well is not surprising (Fig 1). Our comparison of locomotion-induced fluorescence increases with responses to combined locomotion and visual stimulation even suggests that these indicators operate in a similar portion of their dynamic Ca^2+^ sensing range (Fig 2). Microdomain astrocyte Ca^2+^ dynamics and the kinetically related Ca^2+^ waves along processes are at any location short and relatively weak events, more similar to a neuronal response to one or a few action potentials. It is then comprehensible that GCaMP6f was more sensitive at detecting these faint signals than GCaMP3 (Figs 3 and 4) [16].

Locomotion-induced astrocyte Ca^2+^ elevations measured with GCaMP6f reached the peak earlier than GCaMP3 signals (Fig 1F and 1H). Had the earlier rise in the GCaMP6f signal occurred due to increased Ca^2+^ sensitivity or affinity, we would have also expected a longer lasting GCaMP6f fluorescence signal during the Ca^2+^ decay phase, and as a consequence an increased area under the normalized fluorescence trace. However, in our experiments we found that the area under the normalized GCaMP6f fluorescence trace was smaller than for GCaMP3. This happened through a combination of a faster rise and a trend towards a faster decay (Fig 1E and 1G). This observation was consistent with intrinsically faster kinetics of transition between the fluorescent and non-fluorescent states of GCaMP6f compared to all other GCaMPs [16], and it suggests that GCaMP6f represents the true astrocyte Ca^2+^ transient more accurately. Fast kinetics of a Ca^2+^ indicator are particularly important for studies that aim at understanding cause or consequence in the relationship of astrocyte Ca^2+^ dynamics with local neural processes, such as neuronal activity or blood flow, especially when Ca^2+^ imaging is combined with fast measurements of electrical or morphological dynamics [19,52].

## Conclusions

Our investigations indicate that for relating new studies of behavioral state-dependent global astrocyte Ca^2+^ dynamics using GCaMP6f to published work where GCaMP3 has been the most popular GECI, as long as detailed kinetic analyses are not involved, GCaMP6f and GCaMP3 can be considered equivalent. However, for kinetic studies of Ca^2+^ dynamics as well as studies of localization and frequency of small, fast Ca^2+^ events, we can expect novel insight into the complexity of astrocyte function using GCaMP6f. We have focused our project on the basic comparison of the cytosolic versions of GCaMP3 and GCaMP6f. It should be mentioned that GCaMPs have been targeted to the inner leaflet of the plasma membrane and to intracellular organelles to maximize their exposure to locally confined astrocyte Ca^2+^ events [38,53–55]. Our findings further support the promise that the recently developed transgenic mouse line, which enables Cre recombinase-dependent expression of membrane-tethered GCaMP6f [18], will offer a significant step forward towards understanding astrocyte Ca^2+^ dynamics in awake behaving mice.

## Supporting information

S1 Fig Evaluation of calcium wave quantification. Population data of 1—mean correlation coefficient (r). astrocyte tdTomato (GLAST-CreER(+/-);R26-lsl-tdTomato(+/-), n = 6 mice (43 cells); GCaMP3;IP3R2(-/-), n = 8 mice (52 cells); GCaMP3, n = 9 mice (61 cells); GCaMP6f, n = 11 mice (82 cells). Comparisons, which were not significantly different from one-way ANOVA followed by Bonferroni correction (n.s.), are highlighted. Red lines represent mean ± SEM.
(EPS)

S1 File Data file. Excel file containing values of all data points presented in this manuscript and underlying statistical analysis. For explanation of organization of the data file, see S2 File.
(XLSX)

S2 File Explanations for S1 File. (PDF)
