# Supplementary material for: Comparison of GCaMP3 and GCaMP6f for studying astrocyte Ca2+ dynamics in the awake mouse brain
Source: PLoS One. 2017 Jul 24;12(7):e0181113. doi: 10.1371/journal.pone.0181113 (PMC5524333; doi:10.1371/journal.pone.0181113)
Supplement: S2 File — (PDF) [file pone.0181113.s003.pdf]

Raw Data explanations for S1 File.xlsx

A	Fig 1g	- GCaMP3
B	Fig 1g	- GCaMP6f
D	Text	- GCaMP3, maximum rate of fluorescence increase
E	Text	- GCaMP6f, maximum rate of fluorescence increase
G	Text	- GCaMP3, maximum rate of fluorescence decay
H	Text	- GCaMP6f, maximum rate of fluorescence decay
J	Fig 1h	- GCaMP3
K	Fig 1h	- GCaMP6f
M	Fig 1i	- GCaMP3
N	Fig 1i	- GCaMP6f
P	Fig 2b	- GCaMP3, locomotion normalized to locomotion + visual stimulation
Q	Fig 2b	- GCaMP3, normalized locomotion + visual stimulation
S	Fig 2c	- GCaMP6f, locomotion normalized to locomotion + visual stimulation
T	Fig 2c	- GCaMP6f, normalized locomotion + visual stimulation
V	Fig 2d	- GCaMP3
W	Fig 2d	- GCaMP6f
Y	Fig 3d	- GCaMP3
Z	Fig 3d	- GCaMP6f
AB	Fig 4c	- GCaMP3
AC	Fig 4c	- GCaMP6f
AE	S1 Fig	- 1-(mean r) of GLAST-CreER(+/-);R26-lsl-tdTomato(+/-) mice; statistical comparison with Z, AA and AB
AF	S1 Fig	- 1-(mean r) of GLAST-CreER(+/-);R26-lsl-GCaMP3(+/-);IP3R2(-/-) mice; statistical comparison with Y, AA and AB
AG	S1 Fig	- 1-(mean r) of GLAST-CreER(+/-);R26-lsl-GCaMP3(+/-) mice; statistical comparison with Y, Z and AB
AH	S1 Fig	- 1-(mean r) of GLAST-CreER(+/-);R26-lsl-GCaMP6f(+/-) mice; statistical comparison with Y, Z and AA

for all:

row 15	mean
row 16	SD
row 17	SEM
row 19	Lilliefors test for normal distribution (not normally distributed if <0.05)
row 21/22	employed statistical test and p value, if applicable
